# Phospholipid composition in small airway lining fluid among tunnel construction workers exposed to respirable crystalline silica

**DOI:** 10.1186/s12944-025-02790-5

**Published:** 2025-12-11

**Authors:** Mimmi Leite, Per Larsson, Spela Kokelj, Karl-Christian Nordby, Jose Hernan Alfonso, Hatice Koca, Bente Ulvestad, Dag G Ellingsen, Anna-Carin Olin

**Affiliations:** 1 National Institute of Occupational Health, Pb 5330 Majorstuen, Oslo, N-0304 Norway; 2 University of Oslo, Oslo, Norway; 3 Chalmers Mass Spectrometry Infrastructure, Chalmers University of Technology, Gothenburg, Sweden; 4 Occupational and Environmental Medicine, School of Public Health and Community Medicine, Institute of Medicine, Sahlgrenska Academy, University of Gothenburg, Gothenburg, Sweden

**Keywords:** Occupational exposure, Silica, Lipidomics, Pulmonary surfactant, Lipids

## Abstract

**Objective:**

To explore if the phospholipid composition in the small airway lining fluid differed between a group of tunnel construction workers exposed to respirable crystalline silica (RCS) and a reference group.

**Methods:**

In total, 19 healthy, non-smoking workers under exposure to RCS and 21 unexposed referents from the same construction site were included. The participants underwent a health examination including lung function measurements and collection of exhaled particles (PEx) using the Particles in exhaled air (PExA) method. Analysis of PEx included determination of lipids. In total, 95 lipid species, primarily phospholipids, were determined. Non-parametric analyses (Wilcoxon rank-sum test and quantile regression), principal component analysis (PCA) and orthogonal partial least squares discriminant analysis (OPLS-DA) were used for data analysis.

**Results:**

A difference in mol% of phospholipids between the RCS exposed tunnel construction workers and unexposed referents was observed. On lipid class level there was a higher mol% of sphingomyelin (SM) species among exposed workers compared to referents. Regarding single phospholipid species, higher mol % of phosphatidylcholine (PC) and phosphatidylglycerol (PG) species containing diacyl chains with 18:2 (linoleic acid) and 20:4 (arachidonic acid) fatty acid components were observed in the exposed group. Additionally, lower mol% of saturated PC species were observed among the exposed.

**Conclusions:**

Differences in phospholipid composition in the small airway lining fluid between workers exposed to RCS and a reference group were observed. This indicates a possible impact of RCS exposure on phospholipids in the small airways. However, whether these are linked to health effects is currently not known.

**Supplementary Information:**

The online version contains supplementary material available at 10.1186/s12944-025-02790-5.

## Introduction

Exposure to respirable crystalline silica (RCS) is a well-established risk factor for pulmonary disease, causing reduction in lung function [1], lung cancer [2] and silicosis [3]. Furthermore, exposure to RCS has also been linked to autoimmune diseases, suggesting that RCS may play a role in the onset of inflammation and autoimmunity [4]. Respirable particles containing RCS can reach the small airways and the alveoli and deposit [5–7]. The first-line host immune defence in the alveoli consists of a liquid layer lining, here referred to as small airway lining-fluid, with a barrier function [8]. In the alveoli this layer is composed of pulmonary surfactant enabling reduced surface tension and prevention of alveolar collapse during respiration [9]. In addition, pulmonary surfactant constitutes this first barrier to inhaled material and is important for clearance of toxic material and host defence properties. Lipids account for approximately 90% of pulmonary surfactant by weight, with phospholipids being the dominant lipid-class (~ 80–85%) and 5–10% being neutral lipids, mainly cholesterol [10]. Approximately 10% of the pulmonary surfactant is made up of proteins including specific surfactant proteins (Sp) A, B, C and D [10, 11].

Animal studies linked RCS to alterations of pulmonary phospholipids decades ago [12, 13], and a recent study suggests that phospholipids in the lungs may be important in the progression of silicosis [14]. Respirable crystalline silica is suggested to increase surfactant production and turnover in the lungs of rats and sheep [13, 15]. In humans, an increase in lipid-laden macrophages, foam cells, have been observed in patients with silicosis [16]. However, the knowledge on alterations in the phospholipid composition in pulmonary surfactant during exposure to RCS is limited. Moreover, as methods for detecting early signs of silicosis are missing, identification of changes in the phospholipid-profile may potentially serve as biomarkers for adverse effects of exposure.

In Oslo (Norway), tunnel construction workers from a large infrastructure project using tunnel boring machines (TBMs) were invited to participate in a study aiming to explore the constituents of the small airway lining fluid during exposure to rock dust containing RCS. The exposure assessment of this study has been published [17]. The present study aimed to study lipids, focusing on phospholipids, in samples of small airway lining fluid among workers exposed to RCS and a reference group. Further, the aim was to assess if there is an association between phospholipid composition and/or specific phospholipids in the small airway lining fluid and exposure data.

## Materials and methods

### Study population

The aim was to include 20 exposed workers and 20 referents. Information about the study was given at the construction site and participation was voluntary. All tunnel construction workers on the TBMs were eligible for participation if they were not current smokers and did not have asthma or chronic obstructive pulmonary disease (COPD). The referents were recruited among employees in logistics or administrative work (non-exposed work) based on the same criteria. To ensure that health examinations were performed during a period of ongoing exposure to RCS, the exposed workers had to be available for examination on specific dates corresponding to day seven in their work schedule. The participants could not have any signs of airway infection. Twenty tunnel construction workers and 21 non-exposed referents were included. One exposed worker was excluded due to an airway infection, leaving 19 exposed workers in the study.

### Health examination

All participants answered questionnaires covering their respiratory health, smoking history, as well as brief occupational history and current work tasks. The questionnaires were modified versions of previously published questionnaires [18]. The questionnaire covering respiratory health has been validated [19]. In addition, weight and standing height were recorded. Lung function was assessed with Spirare SPS330 spirometer (Diagnostica, Oslo, Norway) according to ATS/ERS recommendations at the time of examination [20] without reversibility testing. Reference values from the Global Lung function Initiative (GLI) were used to calculate predicted normal values and Z-scores [21].

The sampling of droplets from the small airway lining fluid was conducted using the PExA 2.0 instrument (PExA, Gothenburg, Sweden). These samples are referred to as exhaled particles (PEx). The same physician collected the PEx from all participants. A special breathing manoeuvre, as previously described, was used [22]. In short, the participants inhaled particle filtered air and exhaled into the PExA instrument with the aim to collect 120 ng of PEx. The droplets were collected by impaction on a membrane made of hydrophilic polytetrafluoroethylene (PTFE) (Merck Millipore, FHLC02500). The membrane was split in two and stored at −80 °C until analysis. One half was used for analysis of proteins (Sp-A and albumin) and the other half was used for lipid analysis. The use of the PExA method is described in detail elsewhere [23, 24].

### Laboratory analysis

#### Analysis of proteins

Albumin and SpA were quantified using enzyme-linked immunosorbent assays: Human-SpA ELISA kit (RD191139200R, BioVendor, Brno, Czech Republic) and Human-albumin ELISA kit (E-80AL, Immunology Consultant Laboratory, Newberg, OR, USA) using the same procedures as described by Almstrand et *al* [25]. The coefficient of variation (CV) % was in the range 2.4–4.9 for SpA and 0–6.7 for albumin.

#### Analysis of lipids by mass spectrometry

##### Extraction

Stable isotope internal standard mix (SPLASH ^®^ LIPIDOMIX 330707-1EA, Avanti Polar Lipids, Alabaster, AL, USA) was diluted with methanol and spiked to each sample. Lipids were extracted from the PTFE membrane using 400 µl of solvent, consisting of acetonitrile and isopropanol (ratio 2:1), using a thermomixer (40 °C at 700 rpm). The extraction was repeated with 400 µl of methanol. The two eluates were combined in the LC-vial and solvent was evaporated to dryness under a stream of nitrogen. The remaining lipid film was dissolved in 70 µl of solvent, consisting of acetonitrile and isopropanol (ratio 2:1).

##### Analysis

The extracted lipids were analysed using targeted LC-MSMS methods using the Waters ACQUITY UPLC I-Class chromatography system coupled to an electrospray ionization source on a Waters Xevo TQ-XS triple quadrupole mass spectrometer detector (Milford, MA, USA). Phosphatidylcholines (PCs) were analysed using a reverse phase chromatography method that separates phospholipids based on the fatty acid chains. Glucosylceramide (GlcCer), phosphatidylethanolamine (PE) (including phosphatidylethanolamine ether (PE-O)), lysophosphatidylcholine (LPC), sphingomyelin (SM), phosphatidylglycerol (PG), and phosphatidylinositol (PI) were analysed with HILIC chromatography that separates lipid species based on lipid class (the polar head groups). Multiple reaction monitoring mode was used for mass spectrometry data acquisition. A list of mass transitions and the internal standard used for each analyte is provided in Additional file 1. In total, 95 lipids were identified in the samples. Of these, 91 were phospholipids and four were neutral glycosphingolipids (GlcCers). Because phospholipids were dominant in the samples, “phospholipids” will be used to describe the results from the present study.

##### Quantification

Quantification was based on the ratio of the analyte peak area divided by the peak area of the internal standard (one standard per phospholipid class) multiplied with the spiked mol amount of the standard. Since the GlcCer lipids were not included in the SPLASH mix, the PG class internal standard was used as a surrogate class standard. Type II isotope correction was applied for the PE/PG/PI class of phospholipids using the online correction tool LICAR, which is described by Gao et al. [26].

##### Normalisation of lipid amounts

The mol amount can be normalised to sampled particle mass to get a concentration (*concentration = analyte_mol/PEx_gram*) or expressed as compositional data (*mol% of total sample mol amount)*. For comparison both methods for normalisation have been applied, but results are mainly presented as mol%.

The quality of each run was evaluated from a reference sample that was spiked and extracted at different levels. The reference sample consisted of phospholipids extracted from pooled bronchioalveolar lavages and allows monitoring of method performance over time. For the high quality control (QC) samples (*n* = 3) 94% of lipids had an CV < 30%, for the low QC (*n* = 3) 84% of samples had CV < 30% and for a study sample split in half (*n* = 2) before extraction 80% of lipids had a CV < 30%. Although the uncertainty in collected particle mass measurement is unknown, a linear correlation between particle amount and phospholipid amount has been verified. This indicates that if the same amount of particle mass is collected, the amount of small airway lining fluid collected will be approximately the same, allowing all samples to be analysed at the same dilution.

Due to very low levels and uncertainty in quantification, two phospholipids were removed from the dataset, leaving 93 phospholipids available for analysis. These were categorised in eight main classes. Lipid isomers having same fragmentation masses cannot be separated by the mass detector but can sometimes be separated by the chromatography chemistry, resulting in two peaks at different time points. For example the phospholipid PC(16:0_17:1) was found to show two peaks. Full product ion scans verified that it corresponded to the PC(16:0/17:1) and PC(17:1/16:0), with shifted positions of the fatty acid groups. When two peaks were found the first peak is annotated with an A at the end and the second with a B, for example PC(16:0_17:1)A and PC(16:0_17:1)B.

### Data analysis

The phospholipids are presented as compositional data (mol%). Centred log-ratio (clr) transformation was performed before data analyses [27]. For all analyses the results were comparable to analysis using untransformed data. Thus, presented analyses are based on untransformed phospholipid data.

To examine the phospholipids and identify outliers and potential patterns in phospholipid composition between groups, principal component analysis (PCA) and orthogonal partial least square discriminant analysis (OPLS-DA) were used. These methods reduce the dimensions in the data and are suitable for identifying patterns and groupings of the variables. Unit variance scaling was used for all variables in the PCA and OPLS-DA models. The PCA is unsupervised, as exposure status is not specified in the model and a potential separation between groups is thus not caused by known exposure status. In contrast, in the OPLS-DA the exposure status of the participants is given, and the analysis is therefore described as a supervised analysis.

Differences between the groups on single phospholipid-level were tested with Wilcoxon rank-sum test and quantile regression (median). Analyses were performed for all phospholipids and by class and saturation (saturated/unsaturated). For sensitivity analyses the group of saturated phospholipids were analysed with and without PC(16:0/16:0) which is the most abundant phospholipid in pulmonary surfactant.

All analyses were performed with and without women in the reference group. Other relevant variables included in the regression analyses and the OPLS-DA were body mass index (BMI), smoking status (former smoker/never smoker) and lung function parameters.

Data on exposure levels of RCS were collected from a previous published article [17]. Each worker was assigned an exposure level of RCS depending on work title. Correlation between exposure level and mol% of phospholipids that differed significantly between the exposed group and the referents were tested using Spearman’s rho.

A *P* < 0.05 was considered statistically significant for all statistical analyses. Data analysis was performed in Stata 18 (StataCorp LP, TX, USA) and in SIMCA ^®^17 (Sartorius Stedim Data Analytics AB, Sweden). As this study was explorative, no correction for multiple testing was done.

## Results

The study included 40 non-smoking participants with 13 men and eight women in the reference group and 19 men in the exposed group (Table 1). The exposed group had lower FEV_1_/FVC-ratio, higher percentage of former smokers and higher BMI compared to the reference group. Exposure to RCS estimated for all TBM workers was 58 µg/m^3^ (geometric mean) [17].

**Table 1:** Characteristics of all study participants. Median, minimum, and maximum values or percent of total are presented

	Exposed	Referents
N	19	21
Age, years	42 (25–59)	37 (27–54)
Former smokers, N (%)	6 (32%)	3 (15%)
Surfactant protein A in PEx, wt%	5.1 (1.6–11.6)	4.7 (1.8–14.8)
Albumin in PEx, wt%	4.6 (2.0–7.6)	5.3 (2.5–7.1)
FEV_1_, z-score	−0.4 (−1.8-1.9)	−0.8 (−2.3-1.8)
FVC, z-score	−0.1 (−1.9–5.6)	−0.8 (−2.4–1.2)
FEV_1_/FVC, %	79 (66–88)	84 (70–91)
BMI, kg/m^2^	27 (24–33)	24 (19–30)
RCS exposure*, µg/m^3^	58 (2–1517)	n.a.

*N* Number, *n.a* Not applicable, *wt *% Weight%, *FEV*_1_ Forced expiratory volume in 1 s, *FVC* Forced vital capacity, *PEx* Exhaled particles

*Presented as geometric mean (GM) with minimum and maximum measured values, from Leite et *al.* [17]

The PC class was the most abundant comprising more than 80% of total phospholipid signal, followed by PG at approximately 10%, with the remaining classes below 5% (Table 2). There was a significantly higher percentage of SM among the exposed workers compared to the referents. 

**Table 2:** Median, 25th percentile and 75th percentile of the phospholipid classes determined in PEx samples, expressed as a percentage of total phospholipid signal (mol%)

	Exposed			Referents			
Phospholipid class^a^	Median	25%	75%	Median	25%	75%	*P-*value
GlcCer	0.002	0.001	0.003	0.002	0.001	0.002	0.54
SM	**0.22**	**0.19**	**0.28**	**0.18**	**0.16**	**0.22**	***0.03***
LPC	0.24	0.18	0.45	0.20	0.18	0.24	0.12
PE-O	1.00	0.84	1.27	1.24	0.96	1.52	0.09
PE	1.61	1.46	1.70	1.56	1.48	1.76	0.51
PI	3.21	2.70	4.06	3.19	2.95	3.97	0.71
PG	10.5	9.52	10.9	9.9	9.01	10.7	0.26
PC	83.1	82.5	83.8	83.1	82.6	83.5	0.99

^a^*GlcCer* Glucosylceramid, *SM* Sphingomyelin, *LPC* lysophosphatidylcholine, *PE-O* Phosphatidylethanolamine ether, *PE* Phosphatidylethanolamine, *PI* Phosphatidylinositol, *PG* Phosphatidylglycerol, *PC* Phosphatidylcholine

Significant differences (*P*-value < 0.05) marked in bold

**Fig. 1:**
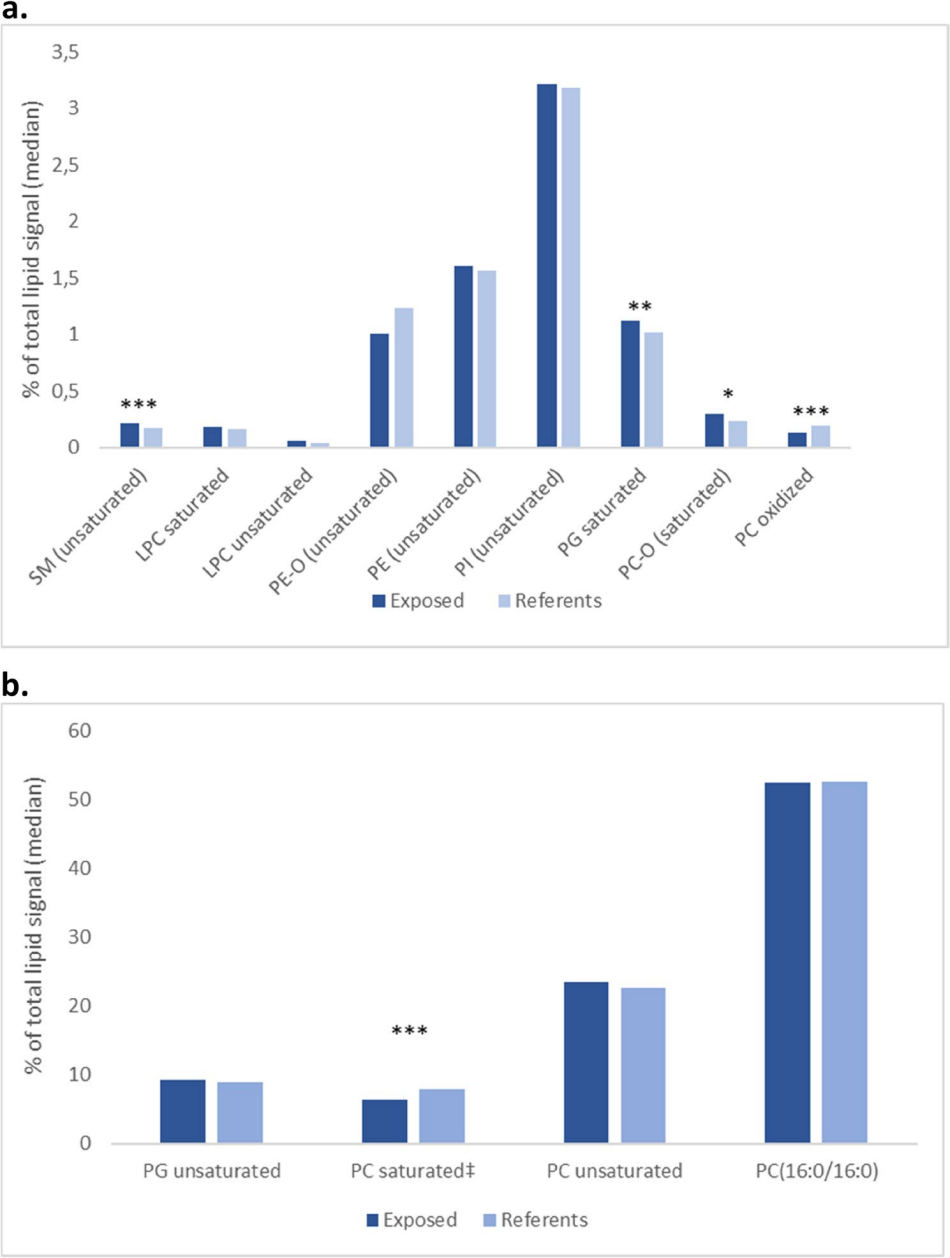
**a** Phospholipids with low abundance (<10 % of total), according to class and saturation, by exposure status. Phospholipid classes with only saturated or unsaturated fatty acids are named by class only and saturation in parentheses. **b** Phospholipids with high abundance (>10 % of total), according to class and saturation, by exposure status. *P<0.05: all participants; **P<0.05: without women; ***P<0.05: all participants and without women. ‡except PC(16:0/16:0). SM: sphingomyelin; LPC: lysophosphatidylcholine; PE-O: phosphatidylethanolamine ether; PE: phosphatidylethanolamine; PI: phosphatidylinositol; PG: phosphatidylglycerol; PC: phosphatidylcholine; PC-O: phosphatidylcholine ether; PC oxidized: oxidized phosphatidylcholine species

Both saturated and unsaturated phospholipids were identified in the LPC, PG, and PC classes. In the PE and PC classes there were groups of ether phospholipids, in addition to oxidized phospholipids identified in the PC class (Fig. 1a & b). The phospholipid PC(16:0/16:0) accounted for over 50% of the phospholipid signal and were similar in the two groups (52.4% in the exposed and 52.5% in the reference group, respectively) (Fig. 1b). The share of saturated PCs excluding PC(16:0/16:0) was lower among the exposed workers versus the referents (Fig. 1b). Further, the oxidized PC phospholipids were significantly lower in the exposed group.

The phospholipid composition in the samples was examined using unsupervised PCA. The model captures the major variance in the dataset and reduces the variables into principal components. Each of the 93 phospholipid species have a loading value that represent the importance of this variable for the calculated principal component. The first PCA model with phospholipid data from all participants uncovered one outlier among the exposed participants (see Additional file 2 for score plot, Figure S1). The phospholipid profile of this participant compared to average for all participants is displayed in a contribution plot in the additional file 2(Additional file 2; Figure S2). This plot showed higher percentage of total for the SM, LPC and GlcCer groups than average for all participants. Further PCA models were made without this participant.

**Fig. 2:**
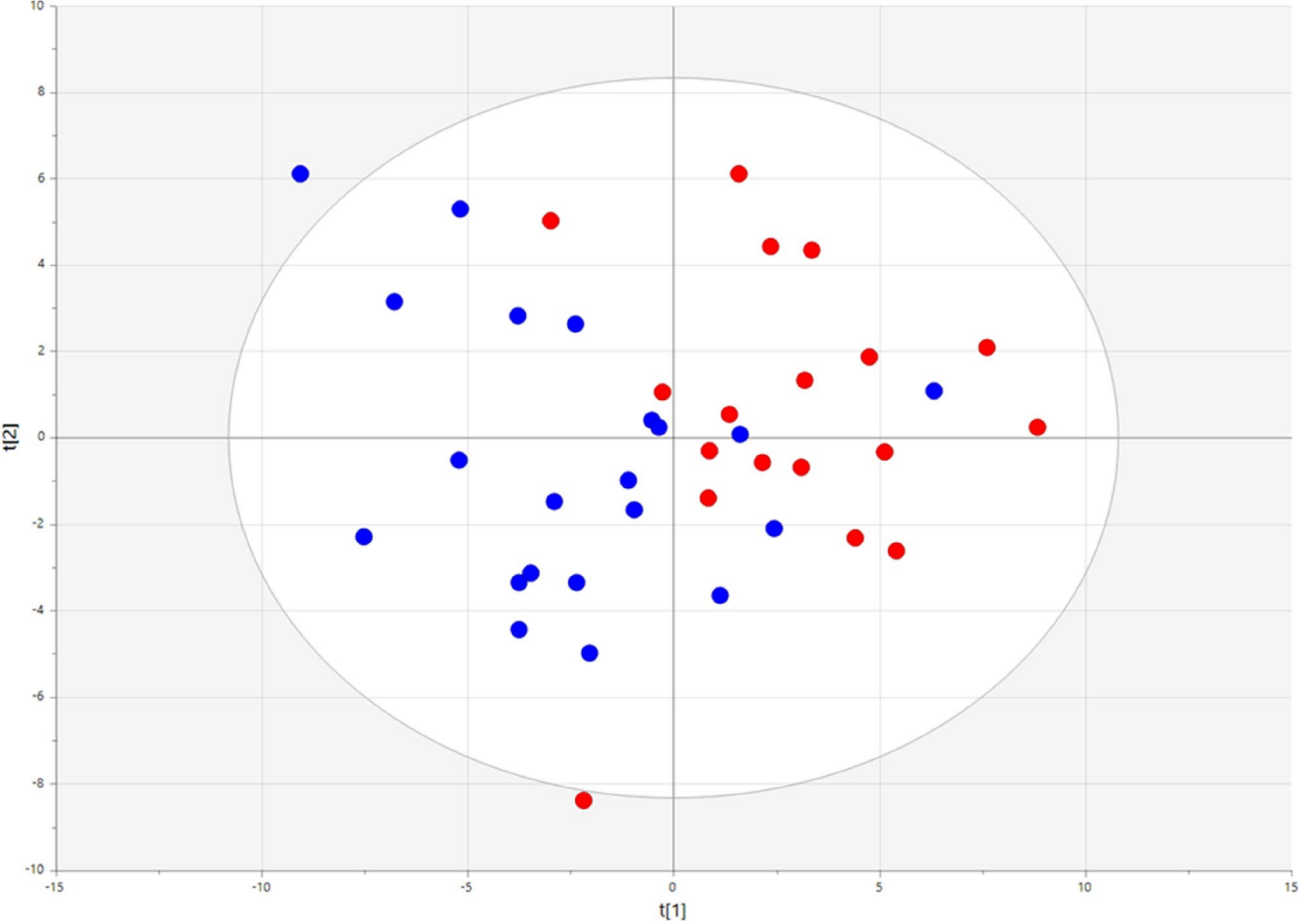
Score plot from unsupervised principal component analysis (PCA) model, showing the first two principal components (first component is horizontal, second is vertical). Coloured by group (red=exposed, blue=referents). Identified outlier from initial PCA excluded

In the score plot of the two first principal components from the final PCA model a separation between the exposed and the reference groups was observed (Fig. 2). The PCA model had an explained variance of 0.47 and a Q2-value of 0.16. The dots in the score plot represent individuals and their phospholipid composition in the PEx samples. The distance between the dots indicates how similar the phospholipid compositions are. Dots in proximity are more similar than those farther apart on the two axes. The corresponding loading plot is shown in Figure S3 (Additional file 2) and identifies the phospholipids contributing the most to the variation described by the first component in the PCA model. As the two study groups differed in BMI, another score plot was used to uncover where the participants were placed according to the level of BMI. No clustering of individuals with similar BMI status was observed (not shown).

**Fig. 3:**
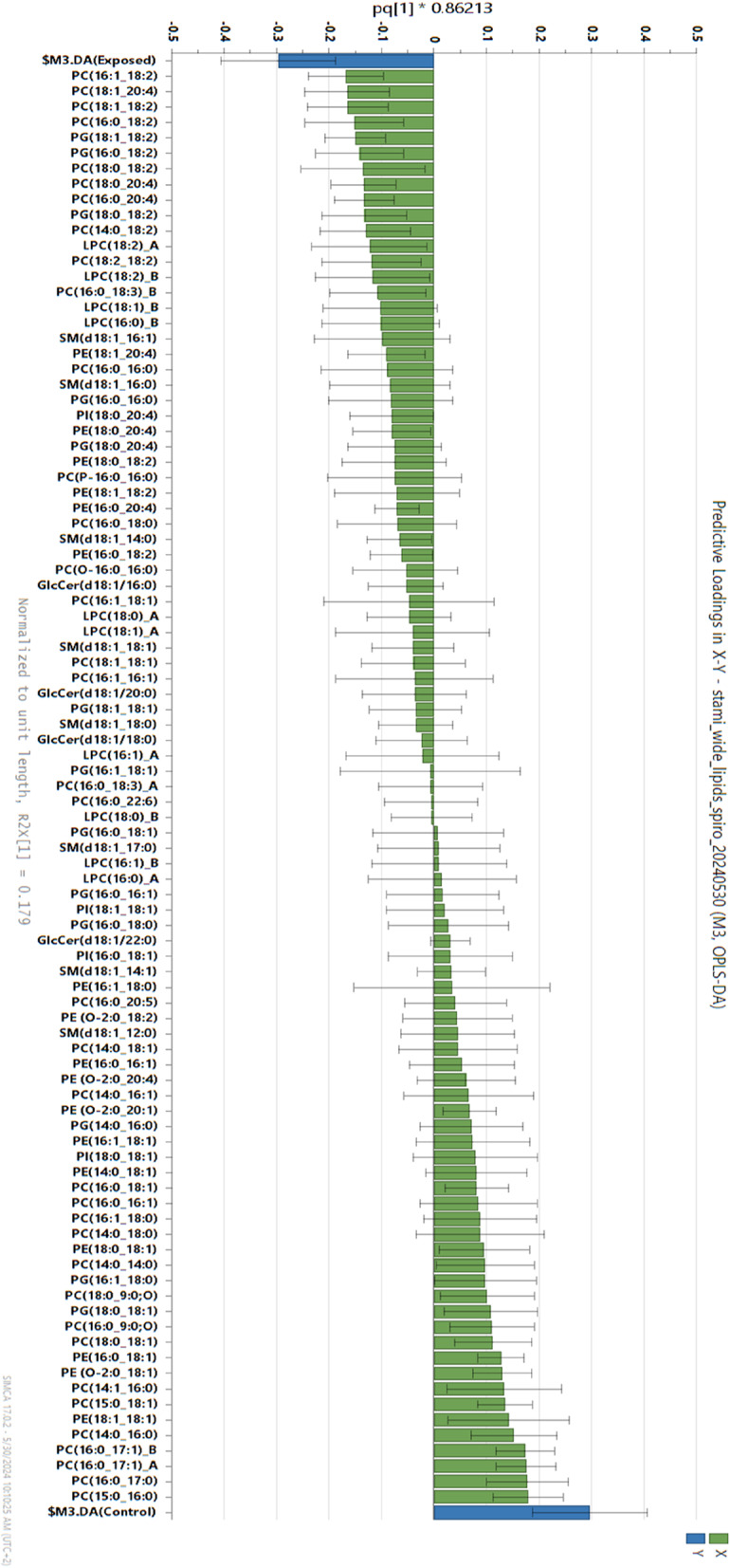
Loading plot from the supervised orthogonal partial least square discriminant analysis (OPLS-DA) comparing the exposed and reference groups according to phospholipid composition in the collected samples. The two blue bars represent the study groups. The exposed on the left and the unexposed on the right side of the plot. Green bars represent the phospholipid loading value which indicates the importance of each phospholipid for the variance in the model. The phospholipids placed at the far ends of the line are the most important for the variance and most associated to the study group at the same end of the line. Lines with whiskers are 95 % confidence intervals

The supervised OPLS-DA model was used to identify the major phospholipid variance between the exposed and the reference group. The loading plot from the OPLS-DA model shown in Fig. 3 describes the phospholipids that were the most influential for the discrimination between the exposed and the referents. The model (R2X = 0.56, Q2 = 0.39) confirmed the pattern seen in the unsupervised PCA model. How important the phospholipids are for the definition of the group are illustrated by the sizes of the loading values (green bars) with 95% confidence intervals, where positive loadings indicate higher proportion of the phospholipid in referents, and negative loadings indicate a higher proportion among the exposed. Species from the PC and PG classes had the strongest influence on both the PCA and OPLS-DA models (Fig. 3 and Figure S3). Additionally, the phospholipids associated with the exposed group, contained the unsaturated FA components 18:2 (linoleic acid) and 20:4 (arachidonic acid). Phospholipids containing the FA component 18:1 (oleic acid) in combination with a saturated FA were lower among exposed than among referents (Fig. 3). According to the model, the saturated phospholipid species PC(15:0_16:0), PC(16:0_17:0), PC(14:0_14:0) and PC(14:0_16:0) were among those with the highest mol% among the referents compared to the exposed group, and the same pattern was true for PC(16:0_17:1) (Fig. 3). Additional info and validation of the PCA and the OPLS-DA models are available in the additional file 2.

**Fig. 4:**
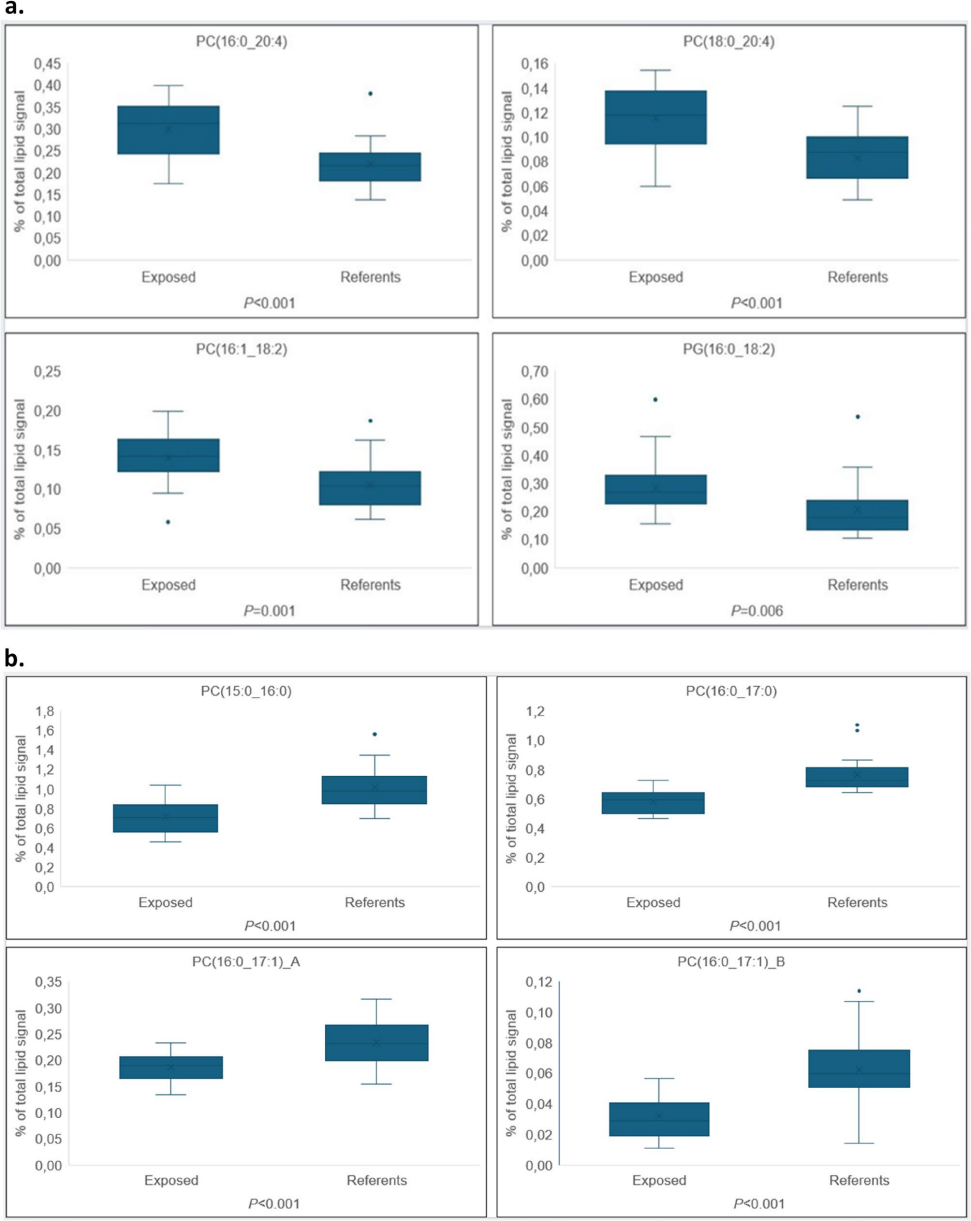
**a** Box plots of phospholipids with higher mol% among the exposed versus the reference group in percent of total phospholipid signal. Horizontal line in boxes, median; boxes, interquartile range; horizontal line at end of whiskers, minimum and maximum values excluding outliers; solid dots above or below boxes; outliers. **b** Box plots of phospholipids with lower mol% among the exposed versus the reference group in percent of total phospholipid signal. Horizontal line in boxes, median; boxes, interquartile range; horizontal line at end of whiskers, minimum and maximum values excluding outliers; solid dots above or below boxes; outliers

Results from the Wilcoxon rank-sum test and quantile regression analyses confirmed the pattern described above (See additional file 2, Table S1). Twenty phospholipids were significantly different between the two groups in both Wilcoxon rank-sum test and quantile regression. Additionally, 21 phospholipids were significantly different in relative abundance in the exposed compared to the reference group in one of the statistical tests with all participants included. In order to visualize some of the significant differences between the groups box plots are presented for a selection of the phospholipids (Fig. 4a and b).

In addition to percentage of total phospholipid signal, the mol/ng PEx concentration was analysed for the two groups (see additional file 2, Table S2). Similar results were observed. Removing women from the models resulted in only small changes, and the observed patterns were still present (Additional file 2; Table S1).

A possible correlation between exposure level of RCS and mol% of phospholipid species were tested, but no correlations were found (not shown).

## Discussion

This study showed different phospholipid composition of the small airways lining fluid among healthy, non-smoking workers exposed to RCS compared to an unexposed reference group. Phospholipid species in the PC and PG classes were the main contributors to these differences. The exposed workers also had a higher share of SM species, a lipid-class with low abundance in pulmonary surfactant [28] and high abundance in cellular membranes [29].

On a single phospholipid-species level it was apparent that exposed workers, compared to the referents, had a higher mol% of phospholipids containing 18:2 and 20:4 FA components (linoleic and arachidonic acids, respectively), while the mol% of phospholipids containing an 18:1 component (oleic acid) in combination with a saturated FA were lower. It was also observed that the exposed workers had lower mol% of saturated PC phospholipids, primarily with one odd-chained FA.

In short, phospholipids consist of a polar head, where an organic molecule (choline, ethanolamine, glycerol, inositol or serine) is linked to a phosphate group, and a tail consisting of two fatty acids (diacyl chain) [10]. Phosphatidylcholine (PC) accounts for the main part of the pulmonary surfactant, with the phospholipid dipalmitoyl phosphatidylcholine (PC(16:0/16:0)) being the main constituent accounting for approximately 50% of PC species, holding the vital function of reducing surface tension in the alveoli [10]. Other components in pulmonary surfactant have specific properties and functions, e.g. phosphatidylglycerol (PG) and phosphatidylinositol (PI) species possessing anti-inflammatory potential [30]. However, it has been suggested that specific functions of phospholipids are not only determined by the class (decided by the head-group), but that also the diacyl chains may decide their function [31].

Alterations in pulmonary surfactant composition have been reported for several diseases such as acute respiratory distress syndrome, COPD, acute lung injury, pneumonia and COVID-19 [11, 32]. It has also been shown that exposure to RCS can cause such alterations in animal studies. Dethloff et al. found an increase in pulmonary phospholipids after instillation of silica in rats [13]. The increase was observed both extracellularly and intracellularly, indicating increased production of pulmonary surfactant in response to silica exposure in non-silicotic rats. A role of phospholipids in silicosis disease progression has also been suggested in a study on mice where altered lipid homeostasis was observed [33]. It has been shown that lipid homeostasis in the alveoli is altered in patients with silicosis, where alveolar macrophages transform into foam cells with an increased uptake of oxidized LDL and an upregulation of CD36 transmembrane receptor suggested to be part of the chain of mechanisms inducing pulmonary fibrosis [16].

Among the findings in the present study on the phospholipid class level were a higher mol% of SM in the exposed group compared to the referents. Sphingomyelin is abundant in cell membranes [34] and can be released into the alveolar space during normal cellular turnover or in response to lung injury and inflammation. Changes in the SM pathways have been suggested in silicosis [35]. The outlier identified in the PCA model had a higher relative abundance of SM than average. However, the difference between the groups remained with this participant excluded. The quantity of SM may alternatively indicate sputum contamination of the PEx sample as sputum holds a high amount of SM [36]. However, no special events were noted during sampling, and the levels of PC(16:0/16:0) were in range with previous PEx samples from small airways.

Further, a lower mol% of oxidized PC species was seen among the exposed workers compared to the referents. The properties of oxidized PC species may be important, such as the protective effect of oxidized PC(16:0_20:4) seen in infection [37]. An increase in the reactive oxygen species (ROS) promoting oxidation is a known part of the pathogenesis leading to silicosis [3], and the results are therefore contradictory to the expected. During PEx sampling the samples are exposed to ambient air, and it is not known how much of the oxidized PC species that may be formed after collection. However, samples from the exposed and referents were handled in the same way.

The observed higher mol % of phospholipid species containing 20:4 FA (arachidonic acid) and 18:2 FA (linoleic acid) among exposed workers in the present study suggest that phospholipids with these FAs may have a function in the response following RCS exposure. The effect of silica exposure on alveolar macrophages in cattle has been studied in vitro, showing increased metabolism of arachidonic acid metabolites like thromboxane, leukotrienes and prostaglandins [38]. The importance of the arachidonic acid pathway is also supported by an in-vitro model, where silica exposed macrophages were shown to induce a pro-inflammatory eicosanoid storm and fibrotic processes [39]. Additionally, results from transcriptomics analysis of human lung tissues showed that the arachidonic acid pathway is up-regulated in silicosis [40]. Regarding linoleic acid; Heeley et al. observed an increase in mol% in phospholipids with 18:2 FA components in asthmatic patients after allergen challenge and suggested that this was linked to an increased infiltration of lipoprotein following lung inflammation [41]. Similar findings have been reported in a study on workers in polymer additive manufacturing [25]. The increase was reported for species in the PG [41] and PC [25, 41] phospholipid classes, consistent with the findings in the present study, suggesting that phospholipids with a linoleic acid component may have a general function following exposures affecting the lungs.

The present study uncovered a lower mol% of PC(15:0_16:0), PC(16:0_17:0) and PC(16:0_17:1) among exposed workers. Odd chained fatty acids like pentadecanoic or heptadecanoic acid (saturated FAs with 15 or 17 carbons, respectively) stems, among other sources, from dairy products and ruminant fat [42], and are indicted as biomarkers of consumption of dairy fat [43]. Due to the odd-chained structure it has been suggested that these FAs cannot be endogenously synthesized. However, alternative routes of synthesis and properties of these FAs have been suggested [42]. It cannot be concluded from the present study whether the difference between the groups for these phospholipids can be attributed to RCS exposure or to nutrition. However, BMI did not affect statistical models.

We cannot conclude on what pathophysiological role the observed patterns in the present study may represent. Both because this study has a cross-sectional design and because the significance of the alterations is difficult to interpret. The differences observed were seen in phospholipids that comprise low levels in the samples and are not among the phospholipids that normally are packed together in pulmonary surfactant. The abundance of phospholipids important for alveolar surface tension like PC(16:0/16:0) were not different between the groups. This indicates that RCS exposure may induce alterations related to other functions of pulmonary surfactant. However, what these functions may be and what role the various phospholipids may have are unknown.

This study presents relative data (mol% of total phospholipid signal). This is an advantage as relative data is not influenced by the uncertainty in the sampled particle mass, or differences in the proportions between proteins and lipids in the samples. However, relative data cannot be used to identify differences in total phospholipid level among the exposed and the referents. The concentrations of phospholipids as *mol_lipid/ng_PEx* were calculated to present an estimate of phospholipid concentrations in the collected samples. The results showed a similar pattern of which phospholipids that were higher and lower in exposed vs. referents as seen for the relative mol%. Calculating phospholipid concentrations based on sampled PEx mass may give a less precise value than the presented mol % of total phospholipid signal as the PEx mass is measured using an optical particle counter that gives an estimate of collected amount of sample. A linear correlation between particle mass and analyte has been demonstrated. However, the precision is not known.

No dose-response correlation between RCS exposure and single phospholipid species was uncovered. The exposure estimates represent the group mean for each work group represented on the tunnel boring machines for a period of two years [17] and may not be representative of the exposure levels of each exposed worker in the present project at the time of sampling as there was a considerable day-to-day variation of exposure levels. However, the exposure assessment indicated above average levels of RCS in the last part of the project period [17], which coincides with the PEx sampling.

This study was restricted to healthy, non-smoking workers and referents based on strict criteria for inclusion in the study. The study was small of size and to remove the interaction between RCS exposure and smoking, restriction to non-smokers was necessary to make the study efficient. The same was considered for the restrictions regarding asthma, COPD, and symptoms of airway infection. Consequently, the results can only be interpreted in the context of the study population; healthy, non-smoking workers. Selection bias cannot be ruled out. The number of available workers exposed to RCS was limited due to a high proportion of smokers on the construction site, as well as logistical constraints related to the scheduling of examinations. No volunteers who met the criteria were excluded from the study. Women were included in the reference group to balance the number of referents against the number of exposed individuals. Our analyses did not change substantially in sensitivity analyses with the reference group restricted to men. However, it would be preferable to include only men in the reference group.

The phospholipids in each class were quantified based on the signal from the internal standard of the lipid class. Not using a matched standard for each phospholipid species can result in over- or underestimation of the true value. This is a limitation when comparing the lipid composition described in the present study to other studies. However, because the over- or underestimation factors are the same in all samples this limitation does not affect the analysis of differences between samples.

## Conclusions

In summary, phospholipid species in samples collected from the small airway lining fluid differed between a group of healthy tunnel construction workers exposed to RCS at a concentration of 58 µg/m^3^ (geometric mean) and an unexposed reference group. Further studies are needed to uncover more about the possible association between RCS exposure and alterations in the phospholipid composition in the small airway lining fluid. It would also be of interest to assess the association between lipid- and protein profiles in PEx and other clinical outcomes, such as small airway function and static lung volumes.

## Supplementary Information

Additional file 1. Additional file 1.xls, A list of mass transitions and the internal standard used for each analyte with corresponding lipid category, lipid class and subclass as provided by LipidMaps (https://www.lipidmaps.org/) 

Additional file 2. Additional file 2.docx, file in .docx format including supplementary figures and tables.

## Data Availability

The data underlying this article cannot be shared publicly due to the privacy of individuals that participated in the study. The data will be shared upon reasonable request to the corresponding author.

## Abbreviations

AM: Alveolar macrophage
ATII cells: Alveolar type II cells
BAL: Bronchoalveolar lavage
BMI: Body mass index
CLR: Centred log-ratio
COPD: Chronic obstructive pulmonary disease
CV: Coefficient of variation
FA: Fatty acid
Glc-Cer: Glucosylceramide
GLI: Global lung function initiative
GM: Geometric mean
ISP: Induced sputum
LPC: Lysophosphatidylcholine
OPLS-DA: Orthogonal partial least squares discriminant analysis
PC: Phosphatidylcholine
PC-O: Ether linked phosphatidylcholine
PCA: Principal component analysis
PE: Phosphatidylethanolamine
PE-O: Ether linked phosphatidylethanolamine
PEx: Exhaled particles
PExA: Particles in exhaled air
PG: Phosphatidylglycerol
PI: Phosphatidylinositol
RCS: Respirable crystalline silica
ROS: Reactive oxygen species
SM: Sphingomyelin
Sp-A,-B,-C,-D: Surfactant protein A, B, C, D
TBM: Tunnel boring machines
QC: Quality control
