# Supplementary material for: Phospholipid composition in small airway lining fluid among tunnel construction workers exposed to respirable crystalline silica
Source: Lipids Health Dis. 2025 Dec 11;24:382. doi: 10.1186/s12944-025-02790-5 (PMC12699859; doi:10.1186/s12944-025-02790-5)
Supplement: Supplementary file 2 — Additional file 2. Additional file 2.docx, file in .docx format including supplementary figures and tables. [file 12944_2025_2790_MOESM2_ESM.docx]

Online supplement

**Phospholipid composition in small airway lining fluid among tunnel construction workers exposed to respirable crystalline silica**

Mimmi Leite^1,2^, Per Larsson^2^, Spela Kokelj^4^, Karl-Christian Nordby^1^, Jose Hernan Alfonso^1^, Hatice Koca^4^, Bente Ulvestad^1^, Dag G Ellingsen^1^, Anna-Carin Olin^4^

^1^ National Institute of Occupational Health, Oslo, Norway

^2^ University of Oslo, Oslo, Norway

^3^ Chalmers Mass Spectrometry Infrastructure, Chalmers University of Technology, Gothenburg, Sweden

^4^ Occupational and Environmental Medicine, School of Public Health and Community Medicine, Institute of Medicine, Sahlgrenska Academy, University of Gothenburg, Gothenburg, Sweden

Address for correspondence: Mimmi Leite, National Institute of Occupational Health,

Pb 5330 Majorstuen, N-0304 Oslo, Norway.

E-mail: mimmi.leitestami.no

**Figure S1** Score plot from unsupervised principal component analysis (PCA) model, showing the first two principal components (first component horizonally, second component vertically). Coloured by group (red=exposed, blue=reference group).

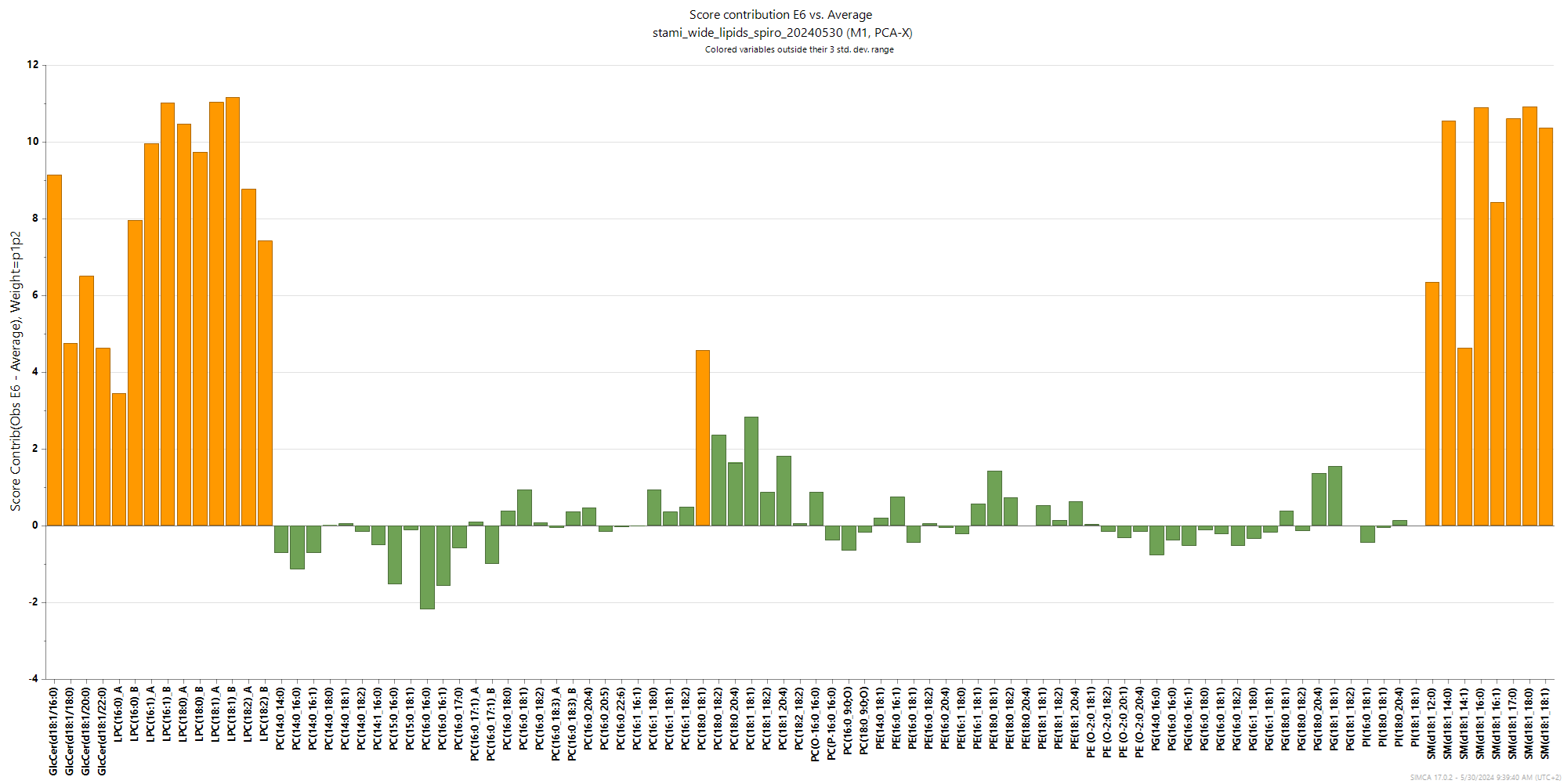
**Figure S2** Contribution plot for identified outlier in PCA model. Score contribution versus average. Variables coloured orange are outside the range of three standard deviation

**Figure S3** Loading plot from unsupervised principal component analysis (PCA) model showing phospholipids associated with the first principal component. The line can be seen in context with horizontal line in the score plot in Figure 2a. Where two phospholipid species have the same name, “A” and “B” are used at the end of the name to differentiate between them.

**Table S1.** Median, 25th percentile and 75th percentile of all lipids expressed as percent of total lipid signal (mol%).

|  | **Exposed** | | | **References** | | |  |
| --- | --- | --- | --- | --- | --- | --- | --- |
| **Lipid name** | Median | 25 % | 75 % | Median | 25 % | 75 % |  |
| **GlcCer(d18:1/16:0)** | 0.001 | ND | 0.001 | 0.001 | 0.0001 | 0.001 |  |
| **GlcCer(d18:1/18:0)** | 0.0003 | 0.00002 | 0.001 | 0.0002 | ND | 0.0004 |  |
| **GlcCer(d18:1/20:0)** | ND | ND | 0.0003 | ND | ND | 0.0002 |  |
| **GlcCer(d18:1/22:0)** | 0.001 | ND | 0.001 | 0.001 | 0.0002 | 0.001 |  |
| **SM(d18:1_12:0)** | 0.0001 | ND | 0.001 | 0.0003 | ND | 0.001 |  |
| **SM(d18:1_14:0)** | 0.013 | 0.011 | 0.015 | 0.011 | 0.0089 | 0.013 |  |
| **SM(d18:1_14:1)** | 0.0001 | ND | 0.001 | 0.0003 | 0.0001 | 0.001 |  |
| **SM(d18:1_16:0)** | 0.17 | 0.14 | 0.21 | 0.13 | 0.12 | 0.17 | ** |
| **SM(d18:1_16:1)** | 0.014 | 0.012 | 0.019 | 0.012 | 0.010 | 0.013 | * |
| **SM(d18:1_17:0)** | 0.0026 | 0.0021 | 0.0036 | 0.0023 | 0.0022 | 0.0029 |  |
| **SM(d18:1_18:0)** | 0.018 | 0.016 | 0.021 | 0.018 | 0.016 | 0.019 |  |
| **SM(d18:1_18:1)** | 0.0041 | 0.0033 | 0.0052 | 0.0038 | 0.0031 | 0.0045 |  |
| **PC(16:0_0:0)** | 0.074 | 0.059 | 0.087 | 0.072 | 0.060 | 0.088 |  |
| **PC(0:0_16:0)** | 0.070 | 0.048 | 0.13 | 0.056 | 0.047 | 0.080 |  |
| **PC(16:1_0:0)_A** | 0.0025 | 0.0013 | 0.0043 | 0.0018 | ND | 0.0036 |  |
| **PC(16:1_0:0)_B** | 0.0005 | ND | 0.002 | ND | ND | 0.001 |  |
| **PC(18:0_0:0)_A** | 0.0019 | ND | 0.0043 | ND | ND | 0.0012 | ** |
| **PC(18:0_0:0)_B** | 0.030 | 0.026 | 0.040 | 0.029 | 0.024 | 0.037 |  |
| **PC(18:1_0:0)_A** | 0.012 | 0.0071 | 0.016 | 0.0098 | 0.0083 | 0.013 |  |
| **PC(18:1_0:0)_B** | 0.010 | 0.0053 | 0.030 | 0.0081 | 0.0067 | 0.0095 |  |
| **PC(18:2_0:0)_A** | 0.012 | 0.0063 | 0.038 | 0.0070 | 0.0060 | 0.014 |  |
| **PC(18:2_0:0)_B** | 0.022 | 0.0054 | 0.10 | 0.011 | 0.0042 | 0.019 | * |
| **PE (O-2:0_18:1)** | 0.46 | 0.34 | 0.57 | 0.60 | 0.49 | 0.76 | * |
| **PE (O-2:0_18:2)** | 0.34 | 0.24 | 0.52 | 0.40 | 0.27 | 0.51 |  |
| **PE (O-2:0_20:1)** | 0.010 | ND | 0.032 | 0.019 | ND | 0.032 |  |
| **PE (O-2:0_20:4)** | 0.24 | 0.20 | 0.26 | 0.26 | 0.21 | 0.34 |  |
| **PE(14:0_18:1)** | 0.0071 | 0.0057 | 0.0010 | 0.0070 | 0.0054 | 0.011 |  |
| **PE(16:0_16:1)** | 0.021 | 0.017 | 0.026 | 0.023 | 0.019 | 0.027 |  |
| **PE(16:0_18:1)** | 0.23 | 0.20 | 0.27 | 0.26 | 0.25 | 0.28 | * |
| **PE(16:0_18:2)** | 0.13 | 0.11 | 0.17 | 0.11 | 0.089 | 0.16 |  |
| **PE(16:0_20:4)** | 0.056 | 0.039 | 0.067 | 0.042 | 0.033 | 0.052 | ** |
| **PE(16:1_18:0)** | ND | ND | 0.0018 | 0.0023 | ND | 0.0050 |  |
| **PE(16:1_18:1)** | 0.020 | 0.014 | 0.030 | 0.022 | 0.019 | 0.026 |  |
| **PE(18:0_18:1)** | 0.17 | 0.16 | 0.19 | 0.17 | 0.17 | 0.20 |  |
| **PE(18:0_18:2)** | 0.10 | 0.094 | 0.12 | 0.091 | 0.073 | 0.10 |  |
| **PE(18:0_20:4)** | 0.056 | 0.045 | 0.068 | 0.047 | 0.033 | 0.055 | * |
| **PE(18:1_18:1)** | 0.42 | 0.37 | 0.47 | 0.48 | 0.45 | 0.54 | *** |
| **PE(18:1_18:2)** | 0.29 | 0.24 | 0.31 | 0.24 | 0.22 | 0.27 |  |
| **PE(18:1_20:4)** | 0.067 | 0.049 | 0.073 | 0.049 | 0.046 | 0.059 |  |
| **PI(16:0_18:1)** | 0.50 | 0.38 | 0.59 | 0.49 | 0.40 | 0.62 |  |
| **PI(18:0_18:1)** | 0.91 | 0.67 | 1.08 | 1.01 | 0.86 | 1.09 |  |
| **PI(18:0_20:4)** | 0.14 | 0.12 | 0.16 | 0.12 | 0.10 | 0.13 | * |
| **PI(18:1_18:1)** | 1.89 | 1.41 | 2.27 | 1.70 | 1.58 | 2.11 |  |
| **PG(14:0_16:0)** | 0.037 | 0.029 | 0.045 | 0.043 | 0.033 | 0.049 |  |
| **PG(16:0_16:0)** | 0.86 | 0.69 | 0.99 | 0.70 | 0.66 | 0.82 | ** |
| **PG(16:0_16:1)** | 0.15 | 0.10 | 0.18 | 0.13 | 0.12 | 0.15 |  |
| **PG(16:0_18:0)** | 0.23 | 0.21 | 0.25 | 0.23 | 0.21 | 0.25 |  |
| **PG(16:0_18:1)** | 2.81 | 2.52 | 3.46 | 2.77 | 2.61 | 2.99 |  |
| **PG(16:0_18:2)** | 0.27 | 0.22 | 0.33 | 0.18 | 0.14 | 0.24 | *** |
| **PG(16:1_18:0)** | 0.093 | 0.072 | 0.11 | 0.10 | 0.083 | 0.13 |  |
| **PG(16:1_18:1)** | 0.15 | 0.12 | 0.18 | 0.14 | 0.12 | 0.17 |  |
| **PG(18:0_18:1)** | 1.70 | 1.58 | 1.96 | 2.08 | 1.68 | 2.18 | ** |
| **PG(18:0_18:2)** | 0.18 | 0.16 | 0.22 | 0.12 | 0.11 | 0.19 | *** |
| **PG(18:0_20:4)** | 0.046 | 0.042 | 0.058 | 0.045 | 0.030 | 0.054 |  |
| **PG(18:1_18:1)** | 3.17 | 2.72 | 3.69 | 2.98 | 2.76 | 3.56 |  |
| **PG(18:1_18:2)** | 0.27 | 0.24 | 0.39 | 0.18 | 0.15 | 0.25 | *** |
| **PC(14:0_14:0)** | 0.11 | 0.087 | 0.19 | 0.17 | 0.11 | 0.19 | ** |
| **PC(14:0_16:0)** | 3.44 | 3.19 | 4.01 | 4.66 | 3.97 | 5.25 | ** |
| **PC(14:0_16:1)** | 0.095 | 0.074 | 0.12 | 0.11 | 0.10 | 0.13 |  |
| **PC(14:0_18:0)** | 0.11 | 0.098 | 0.12 | 0.14 | 0.10 | 0.15 | ** |
| **PC(14:0_18:1)** | 0.35 | 0.29 | 0.42 | 0.381 | 0.34 | 0.42 |  |
| **PC(14:0_18:2)** | 0.071 | 0.057 | 0.084 | 0.056 | 0.047 | 0.065 | *** |
| **PC(14:1_16:0)** | 0.051 | 0.043 | 0.065 | 0.039 | 0.034 | 0.050 | * |
| **PC(15:0_16:0)** | 0.70 | 0.56 | 0.84 | 0.98 | 0.88 | 1.12 | *** |
| **PC(15:0_18:1)** | 0.039 | 0.035 | 0.042 | 0.049 | 0.043 | 0.058 | *** |
| **PC(16:0_16:0)** | 52.4 | 51.5 | 54.0 | 52.5 | 51.0 | 53.3 |  |
| **PC(16:0_16:1)** | 4.82 | 4.68 | 5.65 | 5.50 | 4.92 | 5.89 | ** |
| **PC(16:0_17:0)** | 0.59 | 0.50 | 0.64 | 0.72 | 0.69 | 0.81 | *** |
| **PC(16:0_17:1)_A** | 0.19 | 0.16 | 0.21 | 0.23 | 0.20 | 0.25 | *** |
| **PC(16:0_17:1)_B** | 0.029 | 0.019 | 0.041 | 0.060 | 0.051 | 0.070 | *** |
| **PC(16:0_18:0)** | 1.34 | 1.17 | 1.45 | 1.23 | 1.12 | 1.31 |  |
| **PC(16:0_18:1)** | 7.53 | 7.09 | 7.99 | 7.88 | 7.44 | 8.70 |  |
| **PC(16:0_18:2)** | 3.37 | 2.95 | 3.86 | 2.80 | 2.43 | 3.29 | *** |
| **PC(16:0_18:3)_A** | 0.080 | 0.072 | 0.12 | 0.11 | 0.099 | 0.13 | *** |
| **PC(16:0_18:3)_B** | 0.17 | 0.14 | 0.20 | 0.14 | 0.12 | 0.15 | * |
| **PC(16:0_20:4)** | 0.31 | 0.24 | 0.35 | 0.21 | 0.20 | 0.24 | *** |
| **PC(16:0_20:5)** | 0.025 | 0.018 | 0.032 | 0.031 | 0.025 | 0.041 | * |
| **PC(16:0_22:6)** | 0.020 | 0.013 | 0.029 | 0.022 | 0.018 | 0.026 |  |
| **PC(16:1_16:1)** | 0.14 | 0.12 | 0.15 | 0.13 | 0.11 | 0.15 |  |
| **PC(16:1_18:0)** | 0.18 | 0.17 | 0.20 | 0.19 | 0.17 | 0.22 |  |
| **PC(16:1_18:1)** | 0.30 | 0.26 | 0.35 | 0.28 | 0.24 | 0.32 |  |
| **PC(16:1_18:2)** | 0.14 | 0.12 | 0.16 | 0.10 | 0.083 | 0.12 | *** |
| **PC(18:0_18:1)** | 0.62 | 0.53 | 0.69 | 0.72 | 0.66 | 0.78 | *** |
| **PC(18:0_18:2)** | 0.85 | 0.68 | 1.03 | 0.69 | 0.59 | 0.76 | *** |
| **PC(18:0_20:4)** | 0.12 | 0.095 | 0.14 | 0.088 | 0.067 | 0.099 | *** |
| **PC(18:1_18:1)** | 1.47 | 1.12 | 1.85 | 1.30 | 1.17 | 1.60 |  |
| **PC(18:1_18:2)** | 1.01 | 0.85 | 1.17 | 0.84 | 0.68 | 0.93 | * |
| **PC(18:1_20:4)** | 0.049 | 0.036 | 0.059 | 0.029 | 0.026 | 0.034 | *** |
| **PC(18:2_18:2)** | 0.42 | 0.35 | 0.51 | 0.33 | 0.23 | 0.38 | * |
| **PC(O-16:0_16:0)** | 0.20 | 0.18 | 0.24 | 0.18 | 0.15 | 0.20 | * |
| **PC(P-16:0_16:0)** | 0.10 | 0.031 | 0.13 | 0.054 | 0.031 | 0.10 |  |
| **PC(16:0_9:0al)** | 0.12 | 0.10 | 0.14 | 0.18 | 0.14 | 0.27 | *** |
| **PC(18:0_9:0al)** | 0.015 | 0.012 | 0.016 | 0.020 | 0.016 | 0.024 | *** |

*Significantly difference between groups in Wilcoxon rank-sum test

** Significantly difference between groups in quantile regression

*** Significantly difference between groups in both Wilcoxon rank-sum test and quantile regression, and in sensitivity analysis without women in the control group

ND: not detectable

GlcCer: glucosylceramide (number of carbons : number of double bonds)

PC: phosphatidylcholine (number of carbons : number of double bonds)

PE: phosphatidylethanolamine (number of carbons : number of double bonds)

PI: phosphatidylinositol (number of carbons : number of double bonds)

PG: phosphatidylglycerol (number of carbons : number of double bonds)

PC(16:0_9:0al) and PC(18:0_9:0al): oxidized phosphatidylcholines

**Table S2.** Median, 25th percentile and 75th percentile of all lipids in concentration mol/ng PEx.

|  | **Exposed** | | | **References** | | | Association to exposure  (+/-) |  |
| --- | --- | --- | --- | --- | --- | --- | --- | --- |
| **Lipid name** | Median | 25 % | 75 % | Median | 25 % | 75 % |  |  |
| **GlcCer(d18:1/16:0)** | 3.98E-18 | ND | 4.49E-18 | 2.74E-18 | 2.67E-19 | 3.65E-18 | + |  |
| **GlcCer(d18:1/18:0)** | 8.81E-19 | 4.15E-20 | 1.98E-18 | 1.51E-18 | ND | 2.29E-18 | - |  |
| **GlcCer(d18:1/20:0)** | ND | ND | 1.20E-18 | ND | ND | 8.87E-19 | . |  |
| **GlcCer(d18:1/22:0)** | 2.78E-18 | ND | 3.88E-18 | 2.97E-18 | 1.59E-18 | 3.91E-18 | - |  |
| **SM(d18:1_12:0)** | 5.66E-19 | ND | 2.45E-18 | 1.15E-18 | ND | 2.26E-18 | - |  |
| **SM(d18:1_14:0)** | 5.33E-17 | 3.92E-17 | 6.12E-17 | 5.14E-17 | 4.27E-17 | 6.30E-17 | + |  |
| **SM(d18:1_14:1)** | 5.25E-19 | ND | 2.50E-18 | 1.15E-18 | 3.87E-19 | 1.90E-18 | - |  |
| **SM(d18:1_16:0)** | 7.37E-16 | 6.03E-16 | 8.31E-16 | 6.34E-16 | 5.90E-16 | 7.27E-16 | + |  |
| **SM(d18:1_16:1)** | 6.43E-17 | 4.86E-17 | 7.43E-17 | 5.57E-17 | 4.99E-17 | 6.34E-17 | + |  |
| **SM(d18:1_17:0)** | 1.07E-17 | 8.37E-18 | 1.45E-17 | 1.20E-17 | 9.34E-18 | 1.42E-17 | - |  |
| **SM(d18:1_18:0)** | 7.40E-17 | 6.33E-17 | 9.17E-17 | 8.02E-17 | 7.33E-17 | 9.44E-17 | - |  |
| **SM(d18:1_18:1)** | 1.65E-17 | 1.37E-17 | 2.16E-17 | 1.68E-17 | 1.35E-17 | 2.17E-17 | - |  |
| **PC(16:0_0:0)_A** | 3.54E-16 | 1.81E-16 | 4.02E-16 | 3.51E-16 | 2.29E-16 | 4.30E-16 | + |  |
| **PC(16:0_0:0)_B** | 2.91E-16 | 1.98E-16 | 5.17E-16 | 2.57E-16 | 2.00E-16 | 3.75E-16 | + |  |
| **PC(16:1_0:0)_A** | 1.06E-17 | 4.96E-18 | 1.87E-17 | 9.06E-18 | ND | 1.54E-17 | + |  |
| **PC(16:1_0:0)_B** | 1.47E-18 | ND | 6.97E-18 | ND | ND | 3.80E-18 | + |  |
| **PC(18:0_0:0)_A** | 1.01E-17 | ND | 1.60E-17 | ND | ND | 5.97E-18 | + | ** |
| **PC(18:0_0:0)_B** | 1.22E-16 | 9.88E-17 | 1.81E-16 | 1.30E-16 | 1.02E-16 | 1.85E-16 | - |  |
| **PC(18:1_0:0)_A** | 5.19E-17 | 2.91E-17 | 6.63E-17 | 4.72E-17 | 3.74E-17 | 6.55E-17 | + |  |
| **PC(18:1_0:0)_B** | 4.30E-17 | 2.66E-17 | 1.04E-16 | 3.65E-17 | 2.74E-17 | 5.22E-17 | + |  |
| **PC(18:2_0:0)_A** | 3.57E-17 | 3.13E-17 | 1.21E-16 | 3.15E-17 | 2.39E-17 | 7.94E-17 | + |  |
| **PC(18:2_0:0)_B** | 8.29E-17 | 2.83E-17 | 4.61E-16 | 3.84E-17 | 1.69E-17 | 9.88E-17 | + |  |
| **PE (O-2:0_18:1)** | 1.71E-15 | 1.37E-15 | 2.89E-15 | 3.01E-15 | 2.16E-15 | 3.43E-15 | - | ** |
| **PE (O-2:0_18:2)** | 1.59E-15 | 1.09E-15 | 2.52E-15 | 1.80E-15 | 1.32E-15 | 2.70E-15 | - |  |
| **PE (O-2:0_20:1)** | 4.16E-17 | ND | 1.26E-16 | 8.82E-17 | ND | 1.55E-16 | - |  |
| **PE (O-2:0_20:4)** | 1.02E-15 | 6.92E-16 | 1.32E-15 | 1.28E-15 | 1.03E-15 | 1.42E-15 | - |  |
| **PE(14:0_18:1)** | 3.34E-17 | 1.70E-17 | 3.95E-17 | 3.36E-17 | 2.73E-17 | 4.87E-17 | - |  |
| **PE(16:0_16:1)** | 9.86E-17 | 5.91E-17 | 1.26E-16 | 1.09E-16 | 9.50E-17 | 1.27E-16 | - |  |
| **PE(16:0_18:1)** | 1.04E-15 | 7.31E-16 | 1.29E-15 | 1.29E-15 | 1.04E-15 | 1.38E-15 | - | * |
| **PE(16:0_18:2)** | 5.36E-16 | 3.90E-16 | 6.92E-16 | 5.15E-16 | 4.16E-16 | 7.59E-16 | + |  |
| **PE(16:0_20:4)** | 1.94E-16 | 1.40E-16 | 3.18E-16 | 2.04E-16 | 1.64E-16 | 2.58E-16 | - |  |
| **PE(16:1_18:0)** | ND | ND | 7.98E-18 | 1.14E-17 | ND | 2.51E-17 | - | ** |
| **PE(16:1_18:1)** | 8.41E-17 | 6.14E-17 | 1.11E-16 | 1.05E-16 | 8.76E-17 | 1.23E-16 | - |  |
| **PE(18:0_18:1)** | 6.95E-16 | 5.44E-16 | 8.84E-16 | 8.03E-16 | 7.13E-16 | 1.01E-15 | - |  |
| **PE(18:0_18:2)** | 4.48E-16 | 2.97E-16 | 5.46E-16 | 4.29E-16 | 3.23E-16 | 5.49E-16 | + |  |
| **PE(18:0_20:4)** | 2.33E-16 | 1.79E-16 | 2.84E-16 | 2.19E-16 | 1.72E-16 | 2.55E-16 | + |  |
| **PE(18:1_18:1)** | 1.63E-15 | 1.32E-15 | 2.10E-15 | 2.31E-15 | 1.94E-15 | 2.61E-15 | - | ** |
| **PE(18:1_18:2)** | 1.20E-15 | 8.72E-16 | 1.47E-15 | 1.19E-15 | 9.34E-16 | 1.58E-15 | + |  |
| **PE(18:1_20:4)** | 2.50E-16 | 2.03E-16 | 3.13E-16 | 2.42E-16 | 2.05E-16 | 2.93E-16 | + |  |
| **PI(16:0_18:1)** | 1.88E-15 | 1.28E-15 | 2.77E-15 | 2.21E-15 | 1.97E-15 | 2.58E-15 | - |  |
| **PI(18:0_18:1)** | 3.72E-15 | 2.56E-15 | 5.07E-15 | 4.59E-15 | 3.81E-15 | 5.52E-15 | - |  |
| **PI(18:0_20:4)** | 6.35E-16 | 4.01E-16 | 7.33E-16 | 5.62E-16 | 4.36E-16 | 6.07E-16 | + |  |
| **PI(18:1_18:1)** | 7.03E-15 | 5.95E-15 | 9.56E-15 | 8.57E-15 | 7.34E-15 | 9.36E-15 | - |  |
| **PG(14:0_16:0)** | 1.63E-16 | 1.10E-16 | 2.09E-16 | 2.04E-16 | 1.68E-16 | 2.14E-16 | - | ** |
| **PG(16:0_16:0)** | 3.07E-15 | 2.53E-15 | 4.57E-15 | 3.46E-15 | 3.10E-15 | 3.96E-15 | - |  |
| **PG(16:0_16:1)** | 4.88E-16 | 3.92E-16 | 8.35E-16 | 6.26E-16 | 4.99E-16 | 8.10E-16 | - |  |
| **PG(16:0_18:0)** | 9.86E-16 | 6.80E-16 | 1.20E-15 | 1.07E-15 | 9.65E-16 | 1.18E-15 | - |  |
| **PG(16:0_18:1)** | 1.24E-14 | 9.17E-15 | 1.55E-14 | 1.31E-14 | 1.15E-14 | 1.53E-14 | - |  |
| **PG(16:0_18:2)** | 1.21E-15 | 7.94E-16 | 1.51E-15 | 7.96E-16 | 6.52E-16 | 1.14E-15 | + | ** |
| **PG(16:1_18:0)** | 3.75E-16 | 2.61E-16 | 4.89E-16 | 4.65E-16 | 4.04E-16 | 6.11E-16 | - |  |
| **PG(16:1_18:1)** | 6.16E-16 | 4.52E-16 | 8.01E-16 | 6.61E-16 | 5.40E-16 | 7.62E-16 | - |  |
| **PG(18:0_18:1)** | 7.30E-15 | 4.84E-15 | 9.07E-15 | 9.04E-15 | 8.26E-15 | 1.11E-14 | - | * |
| **PG(18:0_18:2)** | 7.98E-16 | 6.85E-16 | 9.39E-16 | 5.79E-16 | 5.31E-16 | 7.85E-16 | + | ** |
| **PG(18:0_20:4)** | 1.91E-16 | 1.53E-16 | 2.52E-16 | 1.89E-16 | 1.48E-16 | 2.67E-16 | + |  |
| **PG(18:1_18:1)** | 1.21E-14 | 1.02E-14 | 1.69E-14 | 1.41E-14 | 1.26E-14 | 1.67E-14 | - |  |
| **PG(18:1_18:2)** | 1.14E-15 | 1.04E-15 | 1.48E-15 | 9.28E-16 | 6.89E-16 | 1.17E-15 | + |  |
| **PC(14:0_14:0)** | 4.83E-16 | 3.97E-16 | 7.23E-16 | 7.97E-16 | 6.64E-16 | 9.26E-16 | - | ** |
| **PC(14:0_16:0)** | 1.61E-14 | 1.29E-14 | 1.83E-14 | 2.24E-14 | 1.91E-14 | 2.42E-14 | - | ** |
| **PC(14:0_16:1)** | 4.01E-16 | 3.27E-16 | 5.30E-16 | 5.34E-16 | 5.01E-16 | 6.09E-16 | - | ** |
| **PC(14:0_18:0)** | 4.97E-16 | 3.84E-16 | 5.64E-16 | 6.20E-16 | 5.51E-16 | 7.23E-16 | - | ** |
| **PC(14:0_18:1)** | 1.45E-15 | 1.23E-15 | 1.68E-15 | 1.79E-15 | 1.59E-15 | 2.00E-15 | - | ** |
| **PC(14:0_18:2)** | 2.88E-16 | 2.22E-16 | 3.51E-16 | 2.76E-16 | 2.19E-16 | 3.29E-16 | + |  |
| **PC(14:1_16:0)** | 1.92E-16 | 1.33E-16 | 2.02E-16 | 2.51E-16 | 2.10E-16 | 2.93E-16 | - | ** |
| **PC(15:0_16:0)** | 2.95E-15 | 2.23E-15 | 4.12E-15 | 5.12E-15 | 4.08E-15 | 5.37E-15 | - | ** |
| **PC(15:0_18:1)** | 1.72E-16 | 1.18E-16 | 1.97E-16 | 2.44E-16 | 2.09E-16 | 2.90E-16 | - | ** |
| **PC(16:0_16:0)** | 2.31E-13 | 1.76E-13 | 2.53E-13 | 2.55E-13 | 2.19E-13 | 2.66E-13 | - |  |
| **PC(16:0_16:1)** | 2.05E-14 | 1.79E-14 | 2.66E-14 | 2.47E-14 | 2.33E-14 | 2.76E-14 | - | ** |
| **PC(16:0_17:0)** | 2.40E-15 | 1.96E-15 | 3.08E-15 | 3.59E-15 | 3.27E-15 | 3.87E-15 | - | ** |
| **PC(16:0_17:1)_A** | 7.89E-16 | 6.46E-16 | 9.51E-16 | 1.06E-15 | 9.42E-16 | 1.28E-15 | - | ** |
| **PC(16:0_17:1)_B** | 1.36E-16 | 7.47E-17 | 1.67E-16 | 3.21E-16 | 2.54E-16 | 3.48E-16 | - | ** |
| **PC(16:0_18:0)** | 6.12E-15 | 4.02E-15 | 6.41E-15 | 5.57E-15 | 5.30E-15 | 6.41E-15 | - |  |
| **PC(16:0_18:1)** | 3.20E-14 | 2.56E-14 | 3.85E-14 | 3.66E-14 | 3.19E-14 | 4.19E-14 | - |  |
| **PC(16:0_18:2)** | 1.55E-14 | 1.30E-14 | 1.86E-14 | 1.30E-14 | 1.08E-14 | 1.69E-14 | + |  |
| **PC(16:0_18:3)_A** | 3.83E-16 | 2.48E-16 | 5.14E-16 | 5.53E-16 | 4.28E-16 | 6.79E-16 | - | ** |
| **PC(16:0_18:3)_B** | 6.93E-16 | 5.03E-16 | 8.93E-16 | 6.87E-16 | 4.40E-16 | 7.56E-16 | + |  |
| **PC(16:0_20:4)** | 1.27E-15 | 9.65E-16 | 1.50E-15 | 1.01E-15 | 8.36E-16 | 1.19E-15 | + |  |
| **PC(16:0_20:5)** | 9.83E-17 | 6.06E-17 | 1.36E-16 | 1.45E-16 | 1.21E-16 | 1.75E-16 | - |  |
| **PC(16:0_22:6)** | 8.64E-17 | 5.43E-17 | 1.30E-16 | 1.06E-16 | 9.21E-17 | 1.25E-16 | - |  |
| **PC(16:1_16:1)** | 5.87E-16 | 4.31E-16 | 7.30E-16 | 6.36E-16 | 5.16E-16 | 6.79E-16 | - |  |
| **PC(16:1_18:0)** | 7.95E-16 | 5.95E-16 | 9.58E-16 | 8.82E-16 | 8.08E-16 | 9.89E-16 | - |  |
| **PC(16:1_18:1)** | 1.27E-15 | 9.86E-16 | 1.53E-15 | 1.27E-15 | 1.12E-15 | 1.60E-15 | - |  |
| **PC(16:1_18:2)** | 5.91E-16 | 4.67E-16 | 7.15E-16 | 4.57E-16 | 3.58E-16 | 5.78E-16 | + |  |
| **PC(18:0_18:1)** | 2.66E-15 | 1.91E-15 | 3.13E-15 | 3.16E-15 | 2.84E-15 | 3.73E-15 | - | * |
| **PC(18:0_18:2)** | 4.05E-15 | 2.62E-15 | 4.52E-15 | 3.11E-15 | 2.55E-15 | 4.18E-15 | + |  |
| **PC(18:0_20:4)** | 4.80E-16 | 3.68E-16 | 5.93E-16 | 3.78E-16 | 3.15E-16 | 4.43E-16 | + | * |
| **PC(18:1_18:1)** | 6.06E-15 | 5.02E-15 | 7.41E-15 | 6.33E-15 | 5.47E-15 | 7.14E-15 | - |  |
| **PC(18:1_18:2)** | 4.37E-15 | 3.47E-15 | 5.23E-15 | 3.93E-15 | 2.88E-15 | 4.67E-15 | + |  |
| **PC(18:1_20:4)** | 2.05E-16 | 1.80E-16 | 2.35E-16 | 1.38E-16 | 1.07E-16 | 1.94E-16 | + | ** |
| **PC(18:2_18:2)** | 1.94E-15 | 1.51E-15 | 2.35E-15 | 1.59E-15 | 1.01E-15 | 2.19E-15 | + |  |
| **PC(O-16:0_16:0)** | 8.89E-16 | 6.69E-16 | 1.10E-15 | 8.77E-16 | 7.48E-16 | 1.04E-15 | + |  |
| **PC(P-16:0_16:0)** | 4.11E-16 | 1.56E-16 | 5.24E-16 | 2.76E-16 | 1.05E-16 | 4.93E-16 | + |  |
| **PC(16:0_9:0al)** | 5.11E-16 | 4.26E-16 | 6.32E-16 | 8.51E-16 | 5.61E-16 | 1.11E-15 | - | ** |
| **PC(18:0_9:0al)** | 5.71E-17 | 4.53E-17 | 7.59E-17 | 8.59E-17 | 6.84E-17 | 1.32E-16 | - | * |

+ increased concentration of lipid among exposed vs reference

- decreased concentration of lipid among exposed vs reference

*Significantly difference between groups in Wilcoxon rank-sum test

** Significantly difference between groups in quantile regression

ND: not detectable

GlcCer: glucosylceramide (number of carbons : number of double bonds)

SM: sphingomyelin (number of carbons : number of double bonds)

PC: phosphatidylcholine (number of carbons : number of double bonds)

PE: phosphatidylethanolamine (number of carbons : number of double bonds)

PI: phosphatidylinositol (number of carbons : number of double bonds)

PG: phosphatidylglycerol (number of carbons : number of double bonds)

PC(16:0_9:0al) and PC(18:0_9:0al): oxidized phosphatidylcholines

Additional information on PCA and OPLS-DA models

1. Principal component analysis (PCA) model

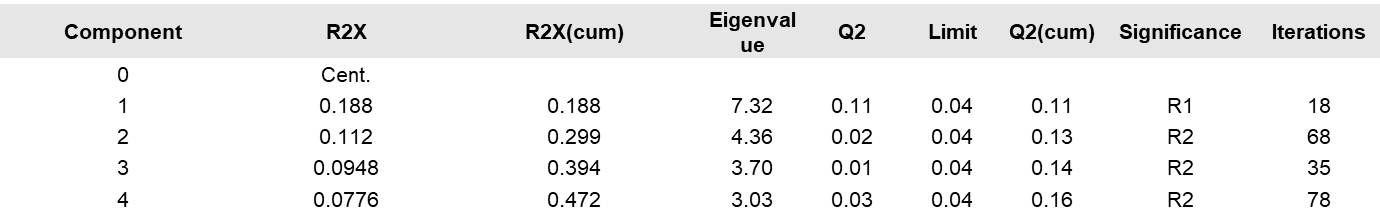


1. OPLS-DA model

| Component | R2X | R2X(cum) | Eigenvalue | R2 | R2(cum) | Q2 | Limit | Q2(cum) | R2Y | R2Y(cum) | EigenvalueY | Significance |
| --- | --- | --- | --- | --- | --- | --- | --- | --- | --- | --- | --- | --- |
| Model |  | 0.18 |  |  | 0.56 |  |  | 0.38 |  | 1 |  |  |
|  |  |  |  |  |  |  |  |  |  |  |  |  |
| Predictive |  | 0.18 |  |  | 0.56 |  |  | 0.38 |  | 1 |  |  |
| P1 | 0.179 | 0.18 | 6.99 | 0.56 | 0.56 | 0.38 | 0.01 | 0.38 | 1 | 1 | 2 | R1 |

1. Validation of OPLS-DA

CV-anova

| **M3** | **SS** | **DF** | **MS** | **F** | **p** | **SD** |
| --- | --- | --- | --- | --- | --- | --- |
| Total corr. | 38 | 38 | 1 |  |  | 1 |
| Regression | 14.6 | 2 | 7.28 | 11.18 | **0.00017** | 2.70 |
| Residual | 23.4 | 36 | 0.65 |  |  | 0.81 |

Permutation validation
